# Proteostasis Dysfunction and Heat Shock Protein Networks in Intervertebral Disc Degeneration: Molecular Mechanisms and Therapeutic Opportunities

**DOI:** 10.3390/cimb48070745

**Published:** 2026-07-21

**Authors:** Zhaoxi Wang, Shijie Chen, Zhaoheng Wang, Yong Sun, Kun Wang, Xuewen Kang

**Affiliations:** 1 Department of Orthopedics, Lanzhou University Second Hospital, Lanzhou 730030, China; wangzhx2024@lzu.edu.cn (Z.W.);; 2 The Second Clinical Medical College, Lanzhou University, Lanzhou 730030, China; 3 Orthopaedics Key Laboratory of Gansu Province, The Second Hospital of Lanzhou University, Lanzhou 730030, China

**Keywords:** heat shock proteins, intervertebral disc degeneration, proteostasis, cellular stress response, therapeutic strategies

## Abstract

Intervertebral disc degeneration is a major pathological contributor to low back pain and functional impairment, yet its complex molecular mechanisms have not been fully elucidated. Heat shock proteins, as important molecular chaperones and core regulators of cellular stress responses, exhibit dual protective and pathogenic roles in the process of intervertebral disc degeneration. This review summarizes the expression changes and related regulatory networks of heat shock protein family members such as HSP70, HSP90, HSP27 and GRP78 in nucleus pulposus and annulus fibrosus cells, comprehensively discussing their involvement in the molecular mechanisms of intervertebral disc degeneration by influencing key processes such as cellular homeostasis, inflammatory responses, apoptosis, autophagy, and the synthesis and degradation of the extracellular matrix. Furthermore, this review highlights HSP-centered proteostasis regulation as an emerging therapeutic framework for IVDD and discusses how HSP modulation may be integrated with biomaterials, physical stimulation, and regenerative strategies. However, direct IVDD-specific evidence for certain HSP members remains limited, and this review also highlights current knowledge gaps and future research directions for HSP-centered proteostasis regulation.

## 1. Introduction

Intervertebral disc degeneration (IVDD) is a progressive process closely associated with various factors such as age, mechanical loads, and genetics. Its hallmark features include nucleus pulposus cell loss, degradation of the extracellular matrix, activation of the inflammatory response, and loss of structural integrity [1,2]. This process not only results in clinical manifestations, predominantly low back pain, but also imposes a significant economic burden on society. The microenvironment of IVDD is highly complex, characterized by various stress conditions such as hypoxia, nutrient deprivation, high osmotic pressure, mechanical stress, and the presence of inflammatory mediators [2,3]. Under these stress conditions, intracellular protein homeostasis faces significant challenges. Heat shock proteins (HSPs), as a class of highly conserved key molecules under stress conditions, critically modulate the fate of intervertebral disc cells and the preservation of extracellular matrix homeostasis [4,5].

HSPs are ubiquitous molecular chaperones, functioning primarily to assist in the correct folding of nascent proteins or those under stress conditions, inhibit their misfolding and aggregation, and play a central role in the protein quality control system [5,6]. Based on molecular weight, HSPs can be classified into several families, including HSP110, HSP90, HSP70, HSP40, and small heat shock proteins (sHSPs) [4,5]. They not only mediate the folding and refolding of nascent proteins but are also widely involved in various biological processes such as signal transduction, apoptosis inhibition, immune regulation, and autophagy [6,7]. In neurodegenerative diseases associated with protein misfolding, HSPs have been confirmed as key cytoprotective factors and potential therapeutic targets [6,8]. However, the specific mechanisms by which HSPs act in IVDD, in the context of associated disrupted protein homeostasis, are not yet fully understood.

In recent years, research has gradually revealed that HSPs play a dual role in IVDD. On the one hand, in the early stages of stress exposure, the upregulation of HSPs serves as a protective response. For example, HSP70 inhibits mitochondrial fission by upregulating SIRT3, thereby alleviating mechanical stress-induced apoptosis of nucleus pulposus cells [9]. The HSP90 inhibitor 17-AAG has been shown to upregulate HSP70 and inhibit the JAK2/STAT3 pathway, reducing inflammation induced by M1-type macrophages in the nucleus pulposus and suppressing disc catabolism [10]. Small heat shock proteins, such as HSPB8, can inhibit abnormal phase transitions and protein aggregation through their chaperone activity. This mechanism has been elucidated in neurodegenerative diseases, suggesting that it may play a similar protective role in IVDD [11,12]. On the other hand, under sustained or excessive stress, the function of HSPs may become dysregulated and even promote pathological processes. For instance, studies have indicated that HSP90 expression levels may be abnormally elevated in IVDD, and its inhibitors can protect nucleus pulposus-derived stem/progenitor cells from stress-induced necroptosis and apoptosis [13]. These findings suggest that the expression and function of HSPs in the microenvironment of degenerated intervertebral discs are dynamically regulated, reflecting a highly complex regulatory network.

Thus, there is significant scientific value in comprehensively elucidating the specific mechanisms of distinct members of the HSP family in IVDD and evaluating the equilibrium they maintain between protecting cell survival and matrix homeostasis versus mediating the inflammatory response and catabolic processes. Furthermore, exploring therapeutic methods targeting HSPs represents an emerging preclinical research direction in IVDD, offering a novel avenue for the development of new biological treatment strategies [6,14]. This narrative review provides a comprehensive overview of IVDD pathogenesis, the structural characteristics and molecular functions of HSPs, and related therapeutic strategies.

### Literature Search Strategy

This narrative review was based on a focused literature search of the PubMed database for articles published between 2000 and 2026. The search terms included combinations of “intervertebral disc degeneration”, “IVDD”, “nucleus pulposus”, “annulus fibrosus”, “proteostasis”, “heat shock protein”, “HSP27”, “HSP40”, “HSP60”, “HSP70”, “HSPA8”, “GRP78”, “HSPA5”, “HSP90”, “molecular chaperone”, “endoplasmic reticulum stress”, “autophagy”, “apoptosis”, “inflammation”, and “extracellular matrix”.

Original research articles, preclinical studies, and human tissue studies reporting the expression, regulation, biological functions, or therapeutic modulation of HSP family members were prioritized. Relevant reviews were used to provide general background on HSP structure, proteostasis, and disease-related mechanisms. Studies directly investigating intervertebral disc cells or tissues were prioritized. Evidence from other diseases was included only when direct IVDD-specific evidence was unavailable and when the reported mechanism was relevant to HSP biology or proteostasis; such evidence was interpreted as indirect or hypothesis-generating. Editorials, conference abstracts, non-English articles without an accessible English abstract, studies lacking sufficient methodological information, and articles unrelated to HSP-mediated proteostasis were excluded. Titles and abstracts were initially screened for relevance, followed by full-text assessment of potentially eligible articles. Because this article is a narrative review rather than a formal systematic review or meta-analysis, no formal risk-of-bias assessment or quantitative evidence synthesis was performed.

## 2. Intervertebral Disc Structure and Degeneration

### 2.1. Structure of the Intervertebral Disc

**Figure 1:**
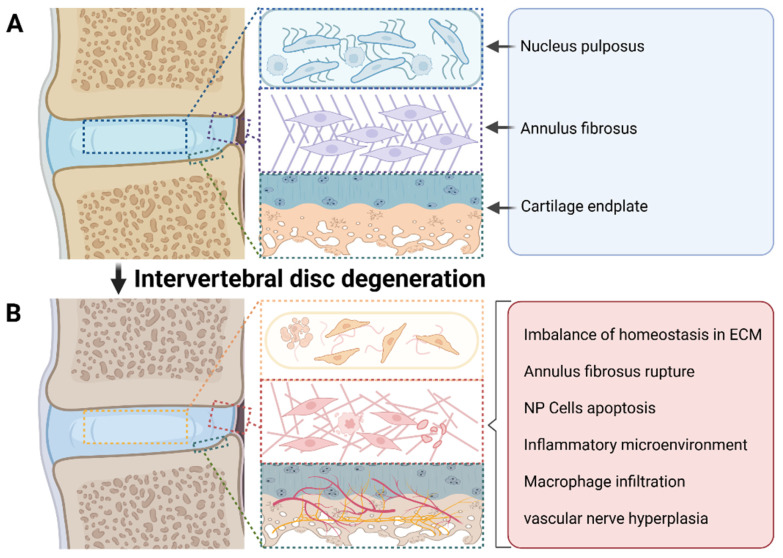
Normal architecture of the intervertebral disc and pathological changes associated with disc degeneration. (**A**): Normal intervertebral disc consists of the nucleus pulposus (NP), annulus fibrosus (AF), and cartilage endplate (CEP). Located centrally, the NP is a hydrated, avascular tissue; the AF surrounds the NP, while the CEP is situated on both superior and inferior aspects of the adjacent vertebral bodies. (**B**): In a degenerative state, the intervertebral disc exhibits pathological changes characterized by disrupted extracellular matrix homeostasis, nucleus pulposus cell apoptosis, annulus fibrosus rupture, macrophage infiltration, and neovascularization. Created in BioRender. Wang, Z. (2026) https://BioRender.com/4i0a6h7 (accessed on 11 July 2026).

The intervertebral disc is situated between adjacent vertebral bodies and is a fibrocartilaginous structure primarily composed of the central nucleus pulposus, the peripheral annulus fibrosus, and the superior and inferior cartilaginous endplates, together forming a closed hydraulic system. The nucleus pulposus is rich in proteoglycans and type II collagen, exhibiting high water content and elasticity, and primarily functions to bear and distribute axial pressure. The annulus fibrosus consists of concentrically arranged lamellae rich in type I collagen fibers, providing tensile strength and torsional stability. The cartilaginous endplate serves as the interface between the intervertebral disc and the vertebral body and functions as the primary conduit for nutrient exchange [15] (Figure 1A).

### 2.2. Pathology of Intervertebral Disc Degeneration

#### 2.2.1. Imbalance in Extracellular Matrix Metabolism and Matrix Degradation

IVDD is characterized by the destructive remodeling of the extracellular matrix, marked by a decrease in proteoglycan content [16], which reduces disc water content and elasticity, consequently compromising its buffering and load-bearing capacity [17]. This loss is the result of an imbalance between anabolism and catabolism, involving increased activity of various matrix-degrading enzymes, such as the upregulation of ADAMTS-5, a key matrix-degrading enzyme [18]. Concurrently, diminished cellular synthetic capacity exacerbates the net loss of matrix [19], ultimately culminating in reduced disc height [17] (Figure 1B).

#### 2.2.2. Dysregulation of Senescence, Apoptosis, and Autophagy in Intervertebral Disc Cells

IVDD is closely associated with cellular senescence in the nucleus pulposus and annulus fibrosus. With increasing age and deterioration of the microenvironment, intervertebral disc cells undergo senescence, characterized by reduced proliferative capacity, functional decline, and the development of a senescence-associated secretory phenotype (SASP) [19]. The SASP comprises pro-inflammatory cytokines, chemokines, growth factors, and matrix-degrading enzymes, which promote persistent inflammation and extracellular matrix degradation [20]. In addition, apoptosis mediated by various signaling pathways such as the mitochondrial pathway and the death receptor pathway leads to a reduction in the number of functional cells, further weakening the repair capacity of the matrix [21]. In degenerated intervertebral discs, the function of autophagy becomes dysregulated. Mitophagy plays a crucial role in maintaining mitochondrial quantity and quality, and its dysfunction is closely related to the initiation and progression of IVDD [22]. In the early stages of degeneration, autophagy activity may be compensatorily enhanced to cope with stress, but in the later stages, it becomes dysfunctional, failing to effectively clear misfolded proteins and damaged organelles, thereby exacerbating cellular dysfunction and death [23]. For example, defects in chaperone-mediated autophagy lead to abnormal accumulation of phospholipase Cγ1, which in turn triggers calcium overload and ultimately induces senescence of nucleus pulposus cells [24] (Figure 1B).

#### 2.2.3. Inflammatory Response and Neurovascular Infiltration

Degenerated intervertebral disc tissue secretes abundant pro-inflammatory cytokines, such as IL-1β and TNF-α, which directly stimulate pain nerve endings and activate matrix-degrading enzymes, forming a vicious cycle [25]. Acid-sensing ion channels are activated in hypoxic environments, thereby contributing to inflammatory responses and pain signal transmission [26]. In the later stages of degeneration, pathological nerve and blood vessel growth occurs in the nucleus pulposus and inner annulus fibrosus, mediated by factors such as VEGF and NGF, which are critical substrates for discogenic pain [27]. Loss of expression of the tendon/ligament-specific marker Tenomodulin accelerates degeneration and triggers angiogenesis and macrophage infiltration [27]. Macrophage infiltration exacerbates local inflammation by polarizing into the pro-inflammatory M1 phenotype and releasing inflammatory factors, ultimately disrupting disc homeostasis [28]. Abnormal infiltration of nerves and blood vessels, combined with persistent inflammation, constitutes key pathological features of pain and structural damage in the late stages of degeneration (Figure 1B).

## 3. Domain Architecture and IVDD-Related Functions of Heat Shock Proteins

**Figure 2:**
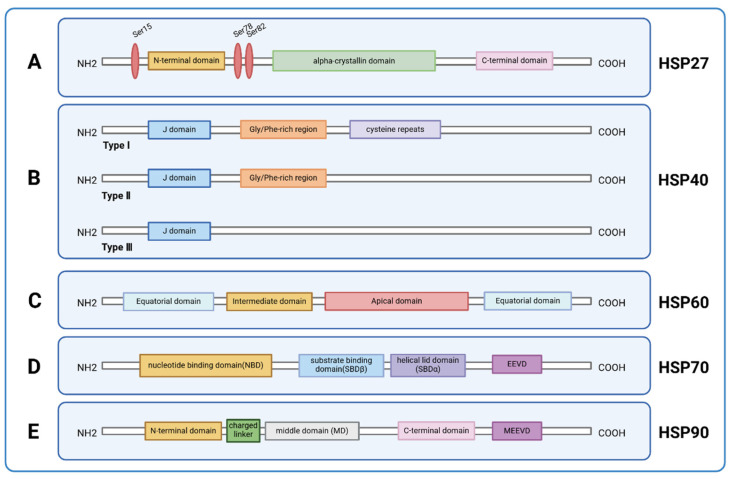
Domain organization of heat shock protein families: (**A**) HSP27; (**B**) HSP40; (**C**) HSP60; (**D**) HSP70; and (**E**) HSP90. HSP27 consists of an N-terminal domain, a C-terminal domain, and an α-crystallin domain, featuring phosphorylation sites at Ser15, Ser78, and Ser82. HSP40 is classified into three types according to the presence or absence of a J domain, a Gly/Phe-rich region, and cysteine repeat sequences. HSP60 is composed of two heptameric rings, totaling 14 subunits, with each subunit comprising an apical domain, an intermediate domain, and an equatorial domain. HSP70 is composed of an N-terminal nucleotide-binding domain (NBD), a substrate-binding domain (SBDβ), a helical lid domain (SBDα), and a C-terminal region containing the conserved charged motif EEVD. HSP90 monomers comprise an N-terminal domain responsible for ATP binding, a middle domain, and a C-terminal domain, with the latter two connected by a charged linker. Created in BioRender. Wang, Z. (2026) https://BioRender.com/7wj9fzk (accessed on 11 July 2026).

Heat shock proteins comprise structurally diverse molecular chaperone families whose domain organization, subcellular localization, and co-chaperone dependence shape their biological functions [29,30,31,32,33]. In IVDD, HSP27/HSPB1, HSP40/DNAJ proteins, HSP60/HSPD1, HSP70 family members, GRP78/HSPA5, and HSP90 isoforms have been implicated to varying degrees in proteostasis maintenance, cellular stress adaptation, apoptosis, autophagy, inflammatory signaling, and extracellular matrix homeostasis [9,10,13,34,35,36,37,38]. However, the strength of supporting evidence differs markedly among individual HSP families. Relatively direct disc-specific evidence is available for HSP70/HSPA8, GRP78/HSPA5, and HSP90, whereas the proposed roles of HSP40 and HSP60 remain largely based on bioinformatic analyses or mechanistic evidence extrapolated from other disease models [1,39,40,41]. Accordingly, this section focuses on the domain architecture and IVDD-related functions of the principal HSP families while distinguishing direct IVDD evidence from indirect or hypothesis-generating findings (Figure 2).

### 3.1. HSP27

#### 3.1.1. Domain Organization

HSP27, also known as HSPB1, belongs to the small heat shock protein family. sHSPs consist of an N-terminal domain (NTD), a C-terminal domain (CTD), and a highly conserved intermediate region known as the α-crystallin domain (ACD). Compared to the ACD, the CTD and NTD of sHSPs are less conserved among different members and species. All three domains play important roles in the function of sHSPs [42]. The functional activity of HSP27 highly depends on its post-transcriptional modification state, particularly phosphorylation. Under non-stress conditions, HSP27 mainly exists as dephosphorylated large molecular oligomers. This conformation confers upon it robust chaperone activity, enabling it to effectively bind and stabilize unfolded or misfolded proteins, thereby maintaining cellular protein homeostasis [43]. However, when cells are subjected to various stress stimuli, HSP27 undergoes phosphorylation at serine residues such as Ser15, Ser78, and Ser82. This process is typically mediated by signaling pathways such as p38 mitogen-activated protein kinase (MAPK) and its downstream effector kinase MAPK-activated protein kinase 2 (MK2) [44]. Phosphorylation causes the dissociation of HSP27 large oligomers into low-molecular-weight dimers or monomers. This conformational change not only alters its chaperone activity but also enables it to translocate from the cytoplasm to the nucleus and modulate various key cell signaling pathways [45] (Figure 2A).

#### 3.1.2. Function

Takao et al. [46] demonstrated that HSP27 is localized in the nuclei of nucleus pulposus cells and chondrocytes, with higher expression in chondrocytes of the cartilaginous endplate during pregnancy and childhood, but its expression decreases with age. Therefore, HSP27 is considered an important molecular marker for the progression of IVDD and may play a key role in cytoprotection. In the pathological environment of IVDD, nucleus pulposus cells are subjected to chronic stress conditions such as hypoxia, nutrient deprivation, and mechanical loading, which may activate kinases such as p38 MAPK/MK2, thereby regulating the phosphorylation state of HSP27 [47]. Studies have shown that alterations in the phosphorylation level of HSP27 are intricately linked to the stability of the cytoskeleton. HSP27 can regulate the spatial organization and cross-linking of cytoskeletal proteins such as vimentin and actin stress fibers through interactions with them, thereby influencing cell morphology, mechanical properties, and differentiation fate [48]. In nucleus pulposus cells of degenerated intervertebral discs, disruption of the cytoskeletal network is a key early event in cellular dysfunction and apoptosis. Phosphorylated HSP27 may affect cell adhesion, migration, and survival by regulating the dynamic reorganization of the cytoskeleton. Furthermore, the phosphorylation state of HSP27 significantly influences cell fate. Phosphorylated HSP27 exerts potent cytoprotective effects via its anti-apoptotic functions by directly inhibiting caspase-3 activation, stabilizing anti-apoptotic proteins, or regulating apoptosis-related signaling pathways such as PI3K/Akt [49].

Ferroptosis has been identified as an important contributor to nucleus pulposus cell dysfunction and IVDD progression [50]. Although HSP27 has been reported to suppress ferroptosis in non-disc cellular models [51], whether HSP27 directly regulates ferroptosis in nucleus pulposus cells remains to be experimentally established. Therefore, further investigation is required to determine how HSP27 expression and phosphorylation influence cytoskeletal integrity, apoptosis, and ferroptosis during IVDD, and to establish whether HSP27 represents a viable therapeutic target in disc degeneration.

### 3.2. HSP40

#### 3.2.1. Domain Organization

HSP40, collectively referred to as the DNAJ protein family, is the largest molecular chaperone family in mammalian cells. All members of this family contain a J domain, which is crucial for their interaction with HSP70 and the activation of HSP70 ATPase activity. Based on domain composition, the HSP40 family is further classified into three major categories: Type I proteins are similar to the Escherichia coli DnaJ protein, containing a J domain, a Gly/Phe-rich region, and cysteine repeats. Type II proteins possess a J domain and a Gly/Phe-rich region but lack cysteine repeats. Type III proteins lack any of the above conserved regions except for the J domain. The protein functions of Type I and Type II proteins appear similar, both capable of binding to non-native substrates. In contrast, Type III proteins may not bind to non-native polypeptides, leading to the speculation that they may lack molecular chaperone function. In addition to the above conserved domains or regions, some DnaJ family members also contain other domains, which may determine the functional diversity of DnaJ proteins [52] (Figure 2B).

#### 3.2.2. Function

The HSP40 protein family serves as crucial co-chaperones for HSP70, constituting a highly synergistic molecular machine alongside HSP70. HSP40 binds to HSP70 via its highly conserved N-terminal J domain [53]. Additionally, HSP40 ensures effective interaction between HSP70 and substrate polypeptides by binding to different sites on the substrate. Moreover, HSP40 stimulates ATP hydrolysis in HSP70 through its J domain, converting HSP70 into an ADP-bound state with increased substrate-binding affinity [53,54]. The HSP70/HSP40 chaperone system mediates diverse cellular processes, including protein folding and prevention of aggregation, stress response, functional protein quality control, and degradation [54]. Certain J-domain proteins also cooperate with mitochondrial HSP70 to facilitate mitochondrial protein import, folding, and biogenesis [55]. These mechanisms provide a potential link between HSP40-dependent proteostasis and mitochondrial homeostasis; however, their relevance to intervertebral disc cells remains to be experimentally validated.

Recently, a study leveraging publicly available gene expression data analyzed the GSE150408 and GSE124272 datasets in the Gene Expression Omnibus (GEO), identifying a total of 1776 differentially expressed genes between IVDD samples and control samples. Weighted gene co-expression network analysis was employed to screen for IVDD-related autophagy genes, leading to the construction and validation of a diagnostic model [1]. DNAJB9, a member of the HSP40 family, was included in this model, suggesting a potential association between HSP40-related chaperone activity, autophagy regulation, and IVDD pathogenesis. However, this evidence is primarily bioinformatic and hypothesis-generating, and the diagnostic or mechanistic relevance of DNAJB9 requires further validation in disc-specific experimental models.

### 3.3. HSP60

#### 3.3.1. Domain Organization

HSP60 is a highly conserved molecular chaperone protein, ubiquitously found across organisms ranging from prokaryotes to eukaryotes. HSP60 is encoded by nuclear genes, synthesized in the cytoplasm, and then transported to specific organelles (mainly mitochondria) to perform its functions. Approximately 80–85% of HSP60 localizes to mitochondria, while the remaining 15–20% resides in the cytoplasm. The chaperonin family to which HSP60 belongs can be divided into Group I chaperonins and Group II chaperonins. HSP60 in bacteria and mitochondria typically belongs to Group I chaperonins, while the TRiC/CCT complex in archaea and eukaryotic cytoplasm belongs to Group II chaperonins. In eukaryotic mitochondria, HSP60 usually consists of two heptameric rings, totaling 14 subunits, with each subunit having a molecular weight of approximately 60 kDa. This double-ring structure forms a central cavity, providing an isolated folding environment for nascent or unfolded proteins, thereby promoting proper protein folding [31]. Each subunit generally includes three functional domains: the apical, intermediate, and equatorial domains. During the folding cycle, HSP60 cooperates with the cofactor HSP10 [56] to undergo ATP-driven conformational changes. Specifically, the apical domain moves upward to expand the cavity, while HSP10 acts as a “lid” that encloses the substrate within a hydrophilic environment, permitting isolated folding. Upon ATP hydrolysis, the lid dissociates, releasing the folded protein (Figure 2C).

#### 3.3.2. Function

HSP60 is not only a key mitochondrial chaperone protein but also an important intracellular and extracellular signaling molecule. Although direct IVDD-specific evidence for HSP60 is currently lacking, studies in non-disc disease models have implicated HSP60 in apoptosis and innate immune signaling. For example, in heart failure, it can regulate cardiomyocyte apoptosis [39], in chronic obstructive pulmonary disease (COPD), it activates the TLR4-MyD88-NF-κB signaling pathway and the NLRP3 inflammasome [40]. Furthermore, it induces autoimmune responses and regulates key signaling pathways [41]. However, these mechanisms of action remain to be directly validated through intervertebral disc-specific studies. These indirect findings support HSP60 as a hypothesis-generating candidate rather than a validated therapeutic target in IVDD.

### 3.4. HSP70

#### 3.4.1. Domain Organization

Members of the HSP70 family comprise at least two of the four structural features characteristic of the prototypical HSP70 (bacterial DnaK): an N-terminal nucleotide-binding domain (NBD) of approximately 45 kDa, linked to a 15 kDa substrate-binding domain (SBDβ), a 10 kDa helical lid domain (SBDα), and a variable-length disordered C-terminal tail. In eukaryotic cytosolic and nuclear HSP70s, the C-terminus of this disordered tail typically harbors a conserved charged motif (glutamate–glutamate–valine–aspartate; EEVD) that mediates interactions with specific cofactors. In the nucleotide-free and ADP-bound states, SBDα docks onto SBDβ, thereby fully enclosing the binding cavity. The NBD and SBDβ are connected by a conserved flexible linker, which is crucial for the allosteric mechanism, enabling the ATP-driven conformational cycling characteristic of the HSP70 chaperone system. This protein further plays a central role in antagonizing apoptosis and senescence, regulating inflammatory responses, and regulating extracellular matrix metabolism [32] (Figure 2D).

#### 3.4.2. Function

The role of HSP70 in IVDD appears to be highly dependent on its isoform and cellular localization. Intracellular HSP70 family members generally support cytoprotection and proteostasis, whereas extracellular HSP70 may act as a damage-associated molecular pattern (DAMP) and amplify inflammatory signaling. However, the strength of direct IVDD-specific evidence differs among these proposed functions.

##### Intracellular HSP70 in Apoptosis and Mitochondrial Homeostasis

**Figure 3:**
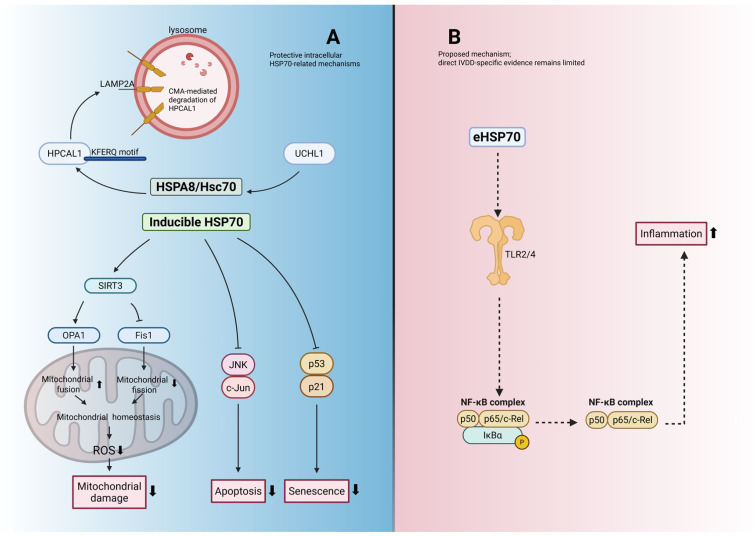
(**A**): Intracellular HSP70 family members exert predominantly protective effects in nucleus pulposus cells. Inducible HSP70 upregulates SIRT3, promotes OPA1 expression, and suppresses Fis1, thereby improving mitochondrial dynamics, maintaining mitochondrial homeostasis, reducing mitochondrial reactive oxygen species production, and attenuating mitochondrial damage. Pharmacological HSP70 activation also suppresses JNK/c-Jun and p53/p21 signaling, resulting in reduced apoptosis and cellular senescence. In parallel, UCHL1-mediated stabilization of HSPA8/Hsc70 facilitates the recognition of KFERQ motif-containing HPCAL1 and its LAMP2A-dependent lysosomal degradation through chaperone-mediated autophagy, thereby limiting nucleus pulposus cell senescence. (**B**): Extracellular HSP70 may function as a damage-associated molecular pattern and is proposed to engage TLR2/4–NF-κB signaling, thereby amplifying inflammatory responses. Dashed arrows indicate a proposed mechanism that is primarily supported by indirect evidence, as direct experimental validation of the extracellular HSP70–TLR2/4–NF-κB axis in IVDD-specific models remains limited. Upward and downward arrows indicate increased and decreased levels or biological effects, respectively. Created in BioRender. Wang, Z. (2026) https://BioRender.com/6yzn70e (accessed on 11 July 2026).

Mechanical loading is an important regulator of mitochondrial homeostasis in nucleus pulposus cells. Cyclic tensile loading has been shown to modulate autophagy through alterations in mitochondrial dynamics, highlighting the close interaction among mechanical stress, mitochondrial quality control, and cellular homeostasis in the intervertebral disc [57]. In a compression-specific cellular model, HSP70 upregulated SIRT3, suppressed Fis1 expression, and promoted OPA1 expression, thereby attenuating mitochondrial fission, reactive oxygen species accumulation, mitochondrial dysfunction, and apoptosis induced by compression [9]. These findings provide direct in vitro evidence that intracellular HSP70 protects nucleus pulposus cells by regulating mitochondrial dynamics. Beyond these disc-specific findings, HSP70 also participates in broader protein quality-control networks through interactions with co-chaperones such as BAG3 and Bis. In a non-disc skeletal muscle model, Bis depletion impaired protein quality control and resulted in the accumulation of damaged proteins, supporting the importance of the HSP70–BAG3/Bis network in cellular proteostasis [58]. However, the contribution of this pathway to intervertebral disc degeneration has not been directly established (Figure 3A).

##### HSPA8-Mediated Chaperone-Mediated Autophagy and Senescence

HSPA8, also known as Hsc70, is a constitutively expressed member of the HSP70 family. HSPA8 is expressed in human lumbar nucleus pulposus tissue, and its expression decreases with increasing severity of disc degeneration [59]. Mechanistically, UCHL1-mediated stabilization of HSPA8 facilitates chaperone-mediated autophagy and promotes the lysosomal degradation of HPCAL1. Because HPCAL1 promotes nucleus pulposus cell senescence, its degradation through the UCHL1/HSPA8/CMA axis reduces senescence-associated β-galactosidase activity and the expression of senescence-associated secretory phenotype factors [34]. These findings provide direct human tissue-associated and in vitro evidence supporting a protective role of HSPA8-mediated CMA in IVDD (Figure 3A).

##### Pharmacological Activation of HSP70

Pharmacological activation of HSP70 with TRC051384 attenuated tert-butyl hydroperoxide-induced apoptosis and senescence in human nucleus pulposus-derived stem/progenitor cells [60]. Mechanistically, HSP70 activation suppressed JNK/c-Jun and p53/p21 signaling, reduced ROS production, and preserved mitochondrial membrane potential and ATP levels. These findings support a protective role of HSP70 under oxidative stress, although in vivo validation remains limited (Figure 3A).

##### HSP70 and Extracellular Matrix Homeostasis

Intracellular HSP70 may indirectly support extracellular matrix homeostasis by maintaining protein-folding capacity, limiting oxidative damage, and preserving nucleus pulposus cell viability. General chaperone biology suggests that HSP70 can facilitate the folding and stability of newly synthesized proteins and stress-sensitive regulatory factors [61,62]. In nucleus pulposus cells, oxidative stress is associated with reduced aggrecan and collagen II expression and increased expression of matrix-degrading enzymes [63]. However, this study did not directly establish HSP70 as the mediator of these matrix-related effects. Therefore, the direct contribution of HSP70 to collagen II and aggrecan synthesis in disc cells remains insufficiently defined.

##### Extracellular HSP70 As a Potential Pro-Inflammatory DAMP

In contrast to the predominantly protective effects of intracellular HSP70, extracellular HSP70 (eHSP70) may function as a damage-associated molecular pattern (Figure 3B). In non-IVDD inflammatory and autoimmune disease models, eHSP70 can interact with immune cells and activate TLR2/4–NF-κB signaling, thereby promoting the production of pro-inflammatory cytokines such as IL-1β, TNF-α, and IL-6 [64,65]. A similar mechanism could theoretically amplify inflammation within the degenerative disc microenvironment. However, direct evidence demonstrating eHSP70-mediated TLR2/4 activation in nucleus pulposus or annulus fibrosus cells remains limited. Therefore, the proposed pathogenic role of eHSP70 in IVDD should currently be regarded as hypothesis-generating rather than experimentally established.

### 3.5. GRP78

#### 3.5.1. Domain Organization

GRP78, also referred to as immunoglobulin heavy chain-binding protein (BiP), is a member of the HSP70 family. Notably, the C-terminus of GRP78 harbors a KDEL (Lys-Asp-Glu-Leu) endoplasmic reticulum retention signal sequence, which ensures that GRP78 is primarily localized in the endoplasmic reticulum lumen and maintains its steady-state localization within this organelle [66].

#### 3.5.2. Function

##### GRP78-Associated ER Stress Under Inflammatory Stimulation

Under conditions of endoplasmic reticulum homeostasis, GRP78 contributes to the maintenance of ER proteostasis. In an IL-1β-induced nucleus pulposus cell degeneration model, GRP78, CHOP, and caspase-12 expression increased, accompanied by reduced cell viability, enhanced apoptosis, and extracellular matrix degradation [35]. Parecoxib attenuated these changes by inhibiting the COX-2/PGE2 pathway, suggesting that GRP78-associated ER stress participates in inflammatory nucleus pulposus cell injury. However, because GRP78 upregulation also represents a general marker of ER stress, its precise causal contribution requires further mechanistic validation.

In addition to the aforementioned pathways, epigenetic modifications, particularly DNA methylation, are closely involved in the regulation of GRP78 and cell fate. In IL-1β-stimulated nucleus pulposus cells, the expression of DNA methyltransferases, including DNMT1 and DNMT3a, is often dysregulated, resulting in aberrant methylation levels within the promoter regions of specific genes. Research indicates that the demethylating agent 5-azacytidine (5-Aza) can inhibit the activity of DNMT1 and DNMT3a, reduce the methylation level of the peroxisome proliferator-activated receptor gamma (PPARγ) gene promoter, and thereby promote PPARγ expression [67]. The upregulation of PPARγ subsequently inhibits GRP78 expression, alleviating IL-1β-induced endoplasmic reticulum stress and nucleus pulposus cell apoptosis.

##### Mechanotransduction and GRP78-Associated ER Stress

Increased extracellular matrix stiffness is an important feature of the degenerative disc microenvironment. In human nucleus pulposus cells, matrix stiffening activates the mechanosensitive ion channel Piezo1, resulting in intracellular calcium overload, ROS accumulation, endoplasmic reticulum stress, cellular senescence, and apoptosis [68]. GRP78 is upregulated as part of the associated ER-stress response; however, whether GRP78 directly mediates the effects of Piezo1 activation on downstream senescence and apoptosis remains to be established.

##### GRP78-Associated ER Stress and NLRP3 Inflammasome Activation in Annulus Fibrosus Cells

In human annulus fibrosus cells, high cyclic stretch induces endoplasmic reticulum stress, accompanied by increased GRP78 expression and activation of the NOX2/ROS/TXNIP/NLRP3 inflammasome pathway. This response promotes IL-1β release and extracellular matrix degradation [36]. Treatment with the endoplasmic reticulum stress inhibitor TUDCA attenuated these changes, supporting ER stress as an upstream contributor to stretch-induced NLRP3 inflammasome activation. Although GRP78 is a prominent marker and molecular chaperone involved in this stress response, additional studies using GRP78-specific genetic or pharmacological interventions are required to establish its direct causal role.

NLRP3 inflammasome activation is not restricted to annulus fibrosus cells and has also been implicated in nucleus pulposus cell degeneration, mitochondrial dysfunction, pyroptosis, and senescence [69]. Collectively, these findings support further investigation of GRP78-associated ER stress and NLRP3 signaling as potential preclinical targets in mechanically induced disc inflammation.

##### Context-Dependent Adaptive Regulation of Extracellular Matrix Synthesis

In addition to its association with maladaptive ER-stress signaling, GRP78 may participate in a context-dependent compensatory response that supports extracellular matrix synthesis. In human nucleus pulposus cells, TNF-α increased nuclear GRP78 expression and promoted circ-4099 transcription. Promoter pull-down and mass spectrometry analyses identified GRP78 as a protein associated with the circ-4099 promoter, whereas GRP78 silencing significantly attenuated TNF-α-induced circ-4099 expression [70]. Circ-4099 subsequently functioned as a competing endogenous RNA for miR-616-5p, thereby relieving the suppression of Sox9 and increasing aggrecan and collagen II expression. These findings suggest that the GRP78/circ-4099/miR-616-5p/Sox9 axis represents a compensatory mechanism that may support extracellular matrix homeostasis during inflammatory stress.

### 3.6. HSP90

#### 3.6.1. Domain Organization

HSP90 functions as a homodimer, and dimerization is crucial for its in vivo function. Each HSP90 monomer consists of three highly conserved domains: the NTD mediates ATP binding, the middle domain (MD) facilitates ATP hydrolysis and client protein binding, and the CTD drives HSP90 dimerization. Its C-terminus contains a Met-Glu-Glu-Val-Asp (MEEVD) motif, which is critical for interacting with co-chaperone proteins containing tetratricopeptide repeat (TPR) domains. The NTD and MD are connected by a long, flexible, charged linker region [71], which regulates NTD–MD contacts and influences HSP90 function [33]. There are two cytoplasmic HSP90 isoforms in mammals, namely the stress-induced HSP90α (HSP90AA1 and HSP90AA2) and the constitutive HSP90β. Although HSP90 isoforms share 85% sequence identity, they have unique functions, such as differential binding to client proteins and their distinct roles in cell differentiation and development [72] (Figure 2E). Notably, most IVDD-related functional studies have used pan-HSP90 inhibitors or have not clearly distinguished between HSP90α and HSP90β. Therefore, the isoform-specific functions of HSP90 in disc degeneration remain unresolved.

#### 3.6.2. Function

Unlike HSP70, which is predominantly cytoprotective in disc cells, HSP90 exhibits a more context-dependent role. By stabilizing different client proteins, HSP90 may support adaptive metabolism under physiological stress but may also maintain inflammatory, necroptotic, and pro-angiogenic signaling complexes during chronic degeneration.

##### HSP90-Mediated Metabolic Adaptation in Nucleus Pulposus Cells

**Figure 4:**
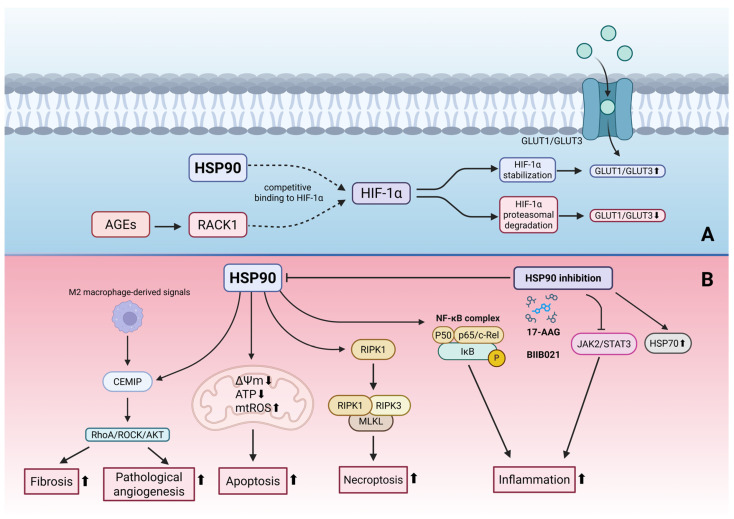
(**A**): Under physiological or adaptive stress conditions, HSP90 competes with receptor for activated C kinase 1 (RACK1) for binding to hypoxia-inducible factor-1α (HIF-1α). HSP90 binding stabilizes HIF-1α and maintains GLUT1/GLUT3 expression, whereas advanced glycation end product-induced RACK1 binding promotes proteasomal degradation of HIF-1α and reduces GLUT1/GLUT3 expression, thereby impairing glucose metabolism in nucleus pulposus cells. (**B**): Under chronic degenerative conditions, HSP90 supports NF-κB-dependent inflammatory signaling, mitochondrial dysfunction and apoptosis, RIPK1/RIPK3/MLKL-mediated necroptosis, and HSP90-dependent CEMIP–RhoA/ROCK/AKT signaling associated with fibrosis and pathological angiogenesis. HSP90 inhibitors, including 17-AAG and BIIB021, suppress HSP90 activity, induce compensatory HSP70 expression, and inhibit JAK2/STAT3 signaling, thereby attenuating inflammatory and cell-death-related responses. Arrows indicate activation or promotion, T-shaped lines indicate inhibition, and dashed lines indicate competitive binding. Upward and downward arrows indicate increased and decreased levels or biological effects, respectively. Created in BioRender. Wang, Z. (2026) https://BioRender.com/s6gxq28 (accessed on 11 July 2026).

Under physiological or early stress conditions, HSP90 exerts cytoprotective effects by stabilizing key proteins and inhibiting misfolding and aggregation through its classic molecular chaperone function, thereby potentially delaying the initiation of degenerative processes. Studies have shown that in nucleus pulposus cells, HSP90 competitively binds to hypoxia-inducible factor-1α (HIF-1α), mitigating the detrimental impact of advanced glycation end products (AGEs) within the intervertebral disc environment [37] (Figure 4A). Under conditions of AGEs accumulation, receptor for activated C kinase 1 (RACK1) competes with HSP90 for binding to HIF-1α, triggering HIF-1α degradation via the proteasome pathway; this cascade downregulates GLUT1/3 expression and ultimately compromises the glucose metabolic function of nucleus pulposus cells. This reveals the critical regulatory role of HSP90 in metabolic stress responses [37].

##### HSP90 in Inflammatory Signaling

HSP90 plays a central regulatory role in inflammation associated with IVDD, primarily by acting as a critical molecular chaperone for the NF-κB signaling pathway. Studies have shown that the HSP90 inhibitor 17-AAG can significantly attenuate the M1-type macrophage-induced inflammatory response in nucleus pulposus cells, partly by inhibiting the NF-κB signaling pathway [10] (Figure 4B). Specifically, HSP90 is crucial for the stability and function of the IKK complex, and inhibiting HSP90 leads to degradation of the IKK complex, thereby blocking the phosphorylation and degradation of IκBα, ultimately inhibiting the nuclear translocation and transcriptional activity of the NF-κB p65 subunit. In the degenerative intervertebral disc microenvironment, levels of inflammatory factors such as IL-1β and TNF-α are significantly elevated. These factors not only directly propagate the inflammatory cascade but may also upregulate HSP90 expression [10]. The upregulated HSP90, in turn, further stabilizes and enhances the transcriptional activity of NF-κB, forming a positive feedback loop that promotes the synthesis of more downstream inflammatory mediators such as cyclooxygenase-2 (COX-2), inducible nitric oxide synthase (iNOS), interleukin-6 (IL-6), and prostaglandin E2 (PGE2), exacerbating inflammation and matrix degradation in the intervertebral disc. Additionally, HSP90 modulates the signal transduction of pattern recognition receptors such as Toll-like receptors, thereby influencing the cellular recognition of damage-associated molecular patterns by intervertebral disc cells, and broadly participating in the initiation and amplification of inflammatory responses.

##### Regulation of Apoptosis and Necroptosis

HSP90 plays a complex regulatory role in the mitochondrial-dependent apoptosis pathway, and its function is closely linked to the fate of nucleus pulposus cells. As a key molecular chaperone, HSP90 directly interacts with pro-apoptotic proteins to significantly regulate mitochondrial outer membrane permeability, a critical step in apoptosis initiation. Studies have shown that HSP90 inhibitors, such as 17-AAG, can significantly suppress stress-induced apoptosis in nucleus pulposus-derived stem/progenitor cells [13]. This protective effect is partially attributed to HSP90’s regulation of mitochondrial function. Inhibiting HSP90 can ameliorate stress-induced mitochondrial dysfunction, including the loss of mitochondrial membrane potential and ATP depletion, while reducing the production of mitochondrial ROS. Beyond its regulation of mitochondrial function, HSP90 also modulates necroptosis in nucleus pulposus cells. Specifically, HSP90 inhibition markedly suppresses necroptosis by modulating the expression and activity of RIPK1, RIPK3, and MLKL [13] (Figure 4B). This indicates that HSP90 is a key molecule stabilizing the necroptosis signaling complex, thereby dictating the activation of downstream pathways originating from death receptors.

##### Fibrosis and Pathological Angiogenesis

In the late stage of IVDD, the pathological repair process is often accompanied by fibrosis and abnormal angiogenesis [73], and HSP90 has also been implicated in fibrotic transformation and pathological angiogenic signaling during IVDD. M2 macrophages play an important role in tissue repair and fibrosis, but their persistent activation can also contribute to pathological alterations. Studies have shown that M2 macrophages may upregulate cell migration-inducing protein (CEMIP) through the HSP90-dependent pathway, thereby promoting the transdifferentiation of NPCs into a fibrotic phenotype and inducing abnormal angiogenesis (Figure 4B). This process is mediated by the RhoA/Rho-associated kinase (ROCK) and protein kinase B (AKT) signaling pathways [38]. The HSP90 inhibitor 17-AAG has been shown to inhibit the expression of CEMIP, thereby ameliorating the fibrotic phenotype of NPCs induced by BMDM-CM; these findings provide disc-specific preclinical evidence that HSP90 inhibition may attenuate fibrotic transformation and pathological angiogenic signaling. However, its long-term efficacy and safety in vivo remain to be established.

##### Compensatory HSP70 Induction After HSP90 Inhibition

The HSP70 family constitutes a core family of protective proteins in the cellular stress response, playing a key role in the pathological process of IVDD by inhibiting apoptosis, regulating autophagy, and maintaining extracellular matrix homeostasis. Under conditions of persistent inflammation or mechanical stress, inhibiting HSP90 function often leads to a compensatory upregulation of HSP70, a critical cellular adaptive mechanism. For example, in nucleus pulposus-derived stem/progenitor cells, treatment with the HSP90 inhibitor BIIB021 significantly reduces compression stress-induced necroptosis and apoptosis, and subsequent analyses suggest that the upregulation of HSP70 contributes to this cytoprotective effect [13]. Similarly, in a model of nucleus pulposus cell inflammation, the HSP90 inhibitor 17-AAG alleviates inflammatory responses and extracellular matrix degradation induced by M1 macrophage-conditioned medium by upregulating HSP70 and inhibiting the JAK2/STAT3 signaling pathway [10] (Figure 4B). Collectively, these findings suggest that compensatory HSP70 induction may contribute to the cytoprotective effects of HSP90 inhibition in disc cells. Nevertheless, this mechanism has been demonstrated predominantly in vitro and requires further validation in animal and human disc models.

##### Translational Limitations and Safety Considerations of HSP90 Inhibition

Although HSP90 inhibitors such as 17-AAG and BIIB021 have shown anti-inflammatory and cytoprotective effects in disc-specific cellular models [10,13], their clinical translation requires careful consideration of safety and dosing limitations. A phase I dose-escalation study of 17-AAG in patients with advanced cancer identified dose-limiting toxicities and schedule-dependent tolerability, indicating that systemic HSP90 inhibition may have a restricted therapeutic window [74]. Although these clinical safety data were obtained in an oncology setting rather than in patients with IVDD, they provide compound-specific evidence regarding the potential limitations of systemic 17-AAG administration. Moreover, because HSP90 maintains the stability and function of numerous physiologically essential client proteins, prolonged or non-selective inhibition may disrupt normal proteostasis and cellular signaling [72]. The optimal dose, treatment duration, isoform selectivity, and therapeutic window of HSP90 inhibitors have not yet been defined in IVDD. Local intradiscal administration or biomaterial-mediated controlled release may provide a potential framework for reducing systemic exposure and improving local drug retention, as illustrated by emerging IVDD-targeted nanofiber and microneedle delivery systems [75,76]; however, burst release, local cytotoxicity, long-term tissue compatibility, and biomechanical safety require careful evaluation. Therefore, HSP90 inhibition should currently be regarded as a preclinical therapeutic approach rather than an established treatment for IVDD (Figure 4B).

The principal HSP family members implicated in IVDD, together with their disc cell or tissue context, experimental models, regulatory mechanisms, biological effects, and evidence categories, are summarized in Table 1. Overall, the available evidence is predominantly derived from in vitro studies, whereas animal, large-animal, and human interventional evidence remains limited.

## 4. HSP-Targeted Molecular Strategies and Translational Opportunities

### 4.1. Mild Photothermal Therapy

Mild photothermal therapy (MPTT) uses photothermal conversion materials and near-infrared irradiation to generate controlled local heating, typically below 45 °C [77]. Unlike conventional high-temperature photothermal therapy, MPTT aims to activate adaptive cellular stress responses while minimizing irreversible thermal injury. In intervertebral disc applications, local temperatures of approximately 40–43 °C have been used to induce heat shock responses without overtly compromising cell viability [75,76]. Heat-induced HSP70 may enhance cellular resistance to oxidative and mitochondrial stress, whereas HSP47 contributes to the folding and maturation of type I and type II collagen.

**Figure 5:**
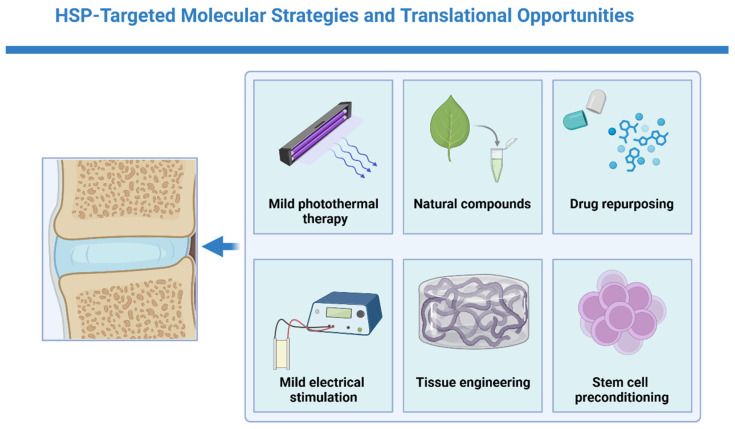
HSP-targeted molecular strategies and translational opportunities in intervertebral disc degeneration. Created in BioRender. Wang, Z. (2026) https://BioRender.com/z5qa0nm (accessed on 11 July 2026).

In IVDD-related and annulus fibrosus repair studies, MPTT has primarily been incorporated into multifunctional biomaterial systems rather than applied as an isolated thermal treatment (Figure 5). A high-strength PDA/GelMA microneedle system loaded with diclofenac sodium enabled annulus fibrosus penetration, NIR-triggered drug release, and local heating. Photothermal stimulation increased HSP-related expression in annulus fibrosus cells, while the combined treatment reduced inflammation and apoptosis, promoted extracellular matrix synthesis, and improved biomechanical parameters in a rat caudal disc injury model [76]. Similarly, a PVA–chitosan nanofibrous membrane containing polyaniline and S-nitrosoglutathione combined MPTT with NIR-controlled nitric oxide release, resulting in increased HSP70 and HSP47 expression, reduced inflammatory and apoptotic responses, and improved extracellular matrix remodeling in vitro and in a rat caudal disc injury model [75]. These findings provide disc-specific preclinical evidence that MPTT may regulate HSP-associated stress responses and serve as an external trigger for controlled therapeutic release. However, because both systems combined heating with drugs, nitric oxide, or other biomaterial functions, the independent contribution of HSP induction remains incompletely defined.

Several translational challenges remain. The penetration depth of 808 nm NIR irradiation may be insufficient for uniform heating of deep lumbar discs, and anatomical differences may result in heterogeneous temperature distribution. Precise thermal monitoring is essential because inadequate heating may fail to activate the desired stress response, whereas excessive or prolonged heating could damage disc cells, cartilaginous endplates, or adjacent neural tissues. The degradation, long-term retention, and local toxicity of photothermal agents also require evaluation; notably, polyaniline has limited in vivo biodegradability and incompletely characterized long-term biosafety [75]. For microneedle systems, puncture-related annulus fibrosus injury, device displacement, mechanical fatigue, and long-term biomechanical effects should also be assessed. Therefore, MPTT should currently be regarded as an experimental biomaterial-assisted strategy for attenuating IVDD-related changes in preclinical models rather than an established method for reversing disc degeneration.

### 4.2. Natural Compounds

Natural compounds have been investigated as modulators of the HSF1–HSP axis and broader proteostasis networks in several experimental systems (Figure 5). Curcumin and resveratrol have been reported to activate HSF1 and increase the expression of downstream heat shock proteins in neurodegenerative disease models, thereby contributing to antioxidant responses, protein quality control, and the clearance of misfolded protein aggregates [78]. Hydroxysafflor yellow A has also been shown to enhance HSF1 and HSP expression and to regulate the unfolded protein response and autophagy in *Caenorhabditis elegans* models [79]. In addition, caffeic acid and luteolin have been associated with increased HSP-16.2 expression and improved stress resistance in non-disc experimental systems [80,81].

These findings suggest that natural compounds may provide a pharmacological basis for modulating HSP-related proteostasis under cellular stress. However, the current evidence is largely indirect and derived from non-IVDD models. Whether these compounds activate comparable HSF1–HSP responses in nucleus pulposus or annulus fibrosus cells has not been clearly established. Their effects on extracellular matrix metabolism, cellular senescence, apoptosis, and inflammatory signaling in disc-specific models also require further investigation. Moreover, limited bioavailability, uncertain intradiscal concentrations, compound-specific off-target effects, and the absence of standardized dosing remain important translational barriers. Therefore, natural HSP modulators should currently be regarded as hypothesis-generating candidates for IVDD rather than established nutritional or therapeutic interventions.

### 4.3. Drug Repurposing

Drug repurposing may provide a practical strategy for identifying HSP- and proteostasis-targeted interventions for IVDD because the pharmacological properties and safety profiles of many candidate compounds have already been partially characterized in other disease settings (Figure 5). The most relevant evidence currently comes from HSP90 inhibitors originally developed for oncology. In disc-specific cellular models, 17-AAG attenuated inflammation and extracellular matrix catabolism induced by M1 macrophage-conditioned medium, accompanied by increased HSP70 expression and inhibition of NF-κB and JAK2/STAT3 signaling [10]. Similarly, BIIB021 reduced compression-induced necroptosis and apoptosis in nucleus pulposus-derived stem/progenitor cells by suppressing RIPK1/RIPK3/MLKL-related signaling, alleviating mitochondrial dysfunction, and inducing compensatory HSP70 expression [13]. These findings provide disc-specific preclinical support for repurposing HSP90 inhibitors to modulate IVDD-related pathological processes, although the available evidence remains predominantly derived from in vitro studies.

Beyond direct HSP90 inhibition, clinically used or previously developed compounds that indirectly regulate HSP-related proteostasis may provide additional disc-specific candidates. Parecoxib attenuated IL-1β-induced apoptosis and extracellular matrix degradation in nucleus pulposus cells, accompanied by suppression of GRP78-, CHOP-, and caspase-12-associated endoplasmic reticulum stress [35]. In human annulus fibrosus cells subjected to high cyclic stretch, the chemical chaperone tauroursodeoxycholic acid attenuated endoplasmic reticulum stress, NLRP3 inflammasome activation, inflammatory cytokine release, and extracellular matrix degradation [36]. In addition, 5-azacytidine reduced endoplasmic reticulum stress and apoptosis in nucleus pulposus cells by preserving PPARγ expression through promoter demethylation [67]. Although these compounds do not directly bind to HSPs, their regulation of GRP78-associated endoplasmic reticulum stress illustrates a broader proteostasis-oriented framework for drug repurposing in IVDD.

Evidence from non-disc disease models may further broaden the range of candidate HSP modulators and reveal additional pharmacological mechanisms. Small-molecule regulation of HSF1 and its downstream heat shock response has been increasingly investigated as a therapeutic strategy in multiple diseases [82]. For example, 17-DMAG has shown antifibrotic activity in a renal fibrosis model [83], whereas quinacrine has been identified as a dual topoisomerase II and HSP90 inhibitor in cancer models [84]. Aspirin-induced HSP70 upregulation has been reported in a *Giardia* model [85], and computational or network-based drug-repurposing studies have identified compounds such as lapatinib and itraconazole as potential modulators of HSP90AA1-associated networks [86]. HSP90 inhibitors have also been reconsidered as antimicrobial agents, although some of these effects may involve mechanisms unrelated to mammalian HSP90 biology, including disruption of bacterial membrane integrity [87]. These findings illustrate the pharmacological diversity of HSP modulation but should be interpreted as indirect and hypothesis-generating evidence rather than proof of therapeutic efficacy in IVDD (Figure 5).

Overall, drug repurposing represents a preclinical strategy for prioritizing candidate compounds rather than an established treatment approach for IVDD. Candidate drugs should first be validated in nucleus pulposus and annulus fibrosus cells, followed by evaluation in animal models, human disc tissues, and, where appropriate, large-animal studies. Particular attention should be given to intradiscal pharmacokinetics, effective local concentrations, isoform selectivity, treatment duration, local cytotoxicity, and systemic adverse effects. Local intradiscal or biomaterial-mediated delivery may help reduce systemic exposure; however, drug retention, release kinetics, long-term tissue compatibility, and biomechanical safety require systematic evaluation.

### 4.4. Mild Electrical Stimulation

Mild electrical stimulation (MES) uses low-intensity electrical currents to modulate cellular and tissue responses. Studies conducted predominantly in non-disc models suggest that electrical stimulation may influence inflammatory responses, wound healing, and pain-related processes [88,89,90]. When combined with heat stimulation, MES has also been associated with activation of the heat shock response. In a diabetic mouse model, combined MES and heat stimulation improved insulin signaling and metabolic abnormalities, accompanied by enhanced HSP expression [91]. Related randomized crossover studies in patients with metabolic syndrome or type 2 diabetes reported improvements in metabolic parameters following the combined intervention [92]. In articular chondrocytes, MES combined with heat stimulation increased HSP70 expression and was associated with enhanced cartilage matrix metabolism [93]. These findings suggest that mild physical stimulation may regulate HSP-associated stress responses in metabolic and musculoskeletal tissues.

However, direct evidence that MES induces protective HSP responses in nucleus pulposus, annulus fibrosus, or cartilaginous endplate cells is currently lacking. Moreover, because most mechanistic studies applied electrical and heat stimulation simultaneously, the independent contributions of electrical stimulation, mild hyperthermia, and HSP induction remain unresolved. Disc-specific studies should therefore compare electrical stimulation alone, heat stimulation alone, and combined treatment, together with appropriate HSP inhibition or knockdown groups. The current intensity, waveform, frequency, treatment duration, electrode configuration, and effective depth of stimulation also require standardization, while potential effects on adjacent neural tissues, cartilaginous endplates, and disc biomechanics should be carefully evaluated. Accordingly, MES should currently be regarded as a hypothesis-generating physical intervention for IVDD rather than an established therapeutic approach.

### 4.5. Tissue Engineering

The avascular and enclosed structure of the intervertebral disc limits systemic drug penetration and makes sustained local treatment challenging. Biomaterial-based delivery systems may therefore provide a means of improving local retention, controlling therapeutic release, and integrating HSP modulation with anti-inflammatory or regenerative interventions. In disc-specific preclinical studies, photothermally responsive microneedle and nanofibrous membrane systems have combined localized heating with controlled delivery of diclofenac sodium or nitric oxide [75,76]. These platforms increased HSP70- and HSP47-related responses, reduced inflammation and apoptosis, and promoted extracellular matrix remodeling in annulus fibrosus cells and rat disc injury models. However, because these systems simultaneously provide thermal stimulation, drug delivery, reactive oxygen species regulation, and structural support, the extent to which their therapeutic effects are specifically mediated by HSP induction remains incompletely defined.

Biomaterials may also offer a conceptual approach for the direct delivery of HSPs or HSP-regulatory molecules. In non-disc disease models, hydrogel microspheres containing TAT-fused HSP70 protected dopaminergic cells and improved functional outcomes in a mouse model of Parkinson’s disease [94]. Similarly, PLGA-based delivery of TAT-HSP27 reduced apoptosis in cardiomyocytes under hypoxic conditions [95]. These studies demonstrate that biomaterial carriers can preserve and deliver biologically active HSP-related proteins. Nevertheless, direct delivery of HSP70, HSP27, or other HSP family members has not yet been adequately evaluated in nucleus pulposus, annulus fibrosus, or cartilaginous endplate models. Therefore, these findings should be regarded as indirect and hypothesis-generating rather than evidence of established efficacy in IVDD.

Several translational challenges require further investigation. HSPs may undergo denaturation, proteolytic degradation, or loss of biological activity during material fabrication, storage, and release. Delivery systems must therefore be optimized for protein stability, loading efficiency, release kinetics, intradiscal retention, cellular uptake, and preservation of chaperone activity. Potential immunogenicity, local cytotoxicity, burst release, biomaterial degradation, and interactions with endogenous proteostasis networks should also be evaluated. In addition, implantation or injection may damage the annulus fibrosus, alter disc biomechanics, or cause material displacement under repetitive loading. Accordingly, HSP-oriented tissue engineering currently represents a preclinical delivery framework whose efficacy and safety require validation in disc-specific cellular models, large-animal studies, and ultimately human tissues.

### 4.6. Stem Cell Preconditioning

The harsh microenvironment of the degenerated intervertebral disc, characterized by hypoxia, nutrient deprivation, hyperosmolarity, inflammatory mediators, and mechanical stress, may substantially impair the survival and function of transplanted mesenchymal stem cells (MSCs). Preconditioning strategies that activate endogenous stress-response pathways before transplantation may therefore warrant investigation as a means of improving cellular resilience. Among these approaches, controlled heat shock has been investigated as a method for inducing HSF1-dependent HSP expression and enhancing the adaptive capacity of stem or progenitor cells [96] (Figure 5).

In non-disc experimental systems, heat shock treatment at 43 °C for 15 min increased intracellular reactive oxygen species in human placenta-derived multipotent cells and activated p38 MAPK and Akt signaling, promoting nuclear translocation of HSF1 and subsequent HSP expression [97]. Heat shock preconditioning has also been applied to bone marrow-derived MSCs. Exosomes released by heat shock-preconditioned BMSCs were enriched in HSP70 and showed greater anti-inflammatory and cytoprotective effects than exosomes from untreated BMSCs in a cisplatin-induced ototoxicity model, accompanied by reduced NLRP3 inflammasome activation [98]. These findings suggest that cellular preconditioning may modify the stress-response profile and paracrine activity of MSCs.

However, direct evidence demonstrating that HSP-oriented stem cell preconditioning improves cell survival, extracellular matrix synthesis, or regenerative efficacy in IVDD models remains limited. The optimal temperature, exposure duration, recovery period, and magnitude of HSP induction may vary among cell sources, and excessive heat shock could induce oxidative injury, senescence, apoptosis, or phenotypic instability. Future studies should compare preconditioned and untreated MSCs in disc-specific hypoxic, hyperosmotic, inflammatory, and mechanically loaded environments, while determining whether the observed effects are directly dependent on HSF1 or specific HSP family members. Variability in cell source, exosome composition, manufacturing consistency, storage stability, dosing, intradiscal retention, and long-term safety must also be addressed. Accordingly, HSP-oriented stem cell preconditioning should currently be regarded as an indirect, hypothesis-generating strategy whose therapeutic relevance requires validation in disc-specific cellular and animal models.

## 5. Challenges—Future Outlook

Although increasing evidence implicates HSP networks in IVDD, several fundamental questions remain unresolved. First, the available evidence is unevenly distributed among HSP families. Relatively direct disc-specific evidence supports the involvement of HSP70/HSPA8, GRP78/HSPA5, and HSP90, whereas the proposed roles of HSP40 and HSP60 remain largely based on bioinformatic analyses or mechanistic extrapolation from non-disc disease models. Moreover, HSP functions are likely to vary according to isoform, subcellular localization, cell type, disease stage, and the duration or intensity of stress. The same HSP family member may support adaptive proteostasis under acute stress but contribute to inflammatory or catabolic signaling under chronic degenerative conditions. Future studies should therefore avoid classifying individual HSPs as uniformly protective or pathogenic and should instead define their context-dependent functions using isoform-specific genetic and pharmacological approaches.

A second priority is to establish the spatiotemporal organization of HSP-related networks within the heterogeneous disc microenvironment. Single-cell RNA sequencing and spatial transcriptomics may help identify HSP expression patterns in nucleus pulposus cells, annulus fibrosus cells, cartilaginous endplate cells, resident progenitor cells, and infiltrating immune cells at different stages of degeneration [38,99]. Integration with proteomics, epigenomics, and spatially resolved protein analysis could further distinguish transcriptional changes from alterations in protein abundance, localization, post-translational modification, and chaperone activity. Such approaches may clarify whether pathways including HSPA8-mediated chaperone-mediated autophagy, HSP90-dependent inflammatory and necroptotic signaling, and GRP78-associated endoplasmic reticulum stress are restricted to specific cellular subpopulations or anatomical regions. Related pathways identified in non-disc stem-cell models, including the Prominin-2/BACH1/GLS axis involved in ferroptosis regulation [100], may also warrant investigation, but their relationship with HSF1 or HSP signaling in disc cells requires direct experimental validation.

Therapeutic development must also account for the bidirectional and network-level functions of HSPs. Rather than globally increasing or suppressing the heat shock response, future strategies may need to enhance protective intracellular chaperone activity while limiting pathogenic extracellular or client-protein-dependent signaling. Disc-specific studies have provided preliminary examples of multifunctional approaches, including photothermally responsive microneedles and nitric oxide-releasing nanofibrous membranes that combine controlled heating with anti-inflammatory or regenerative functions [75,76]. Resveratrol has also shown protective effects in human nucleus pulposus cells through inhibition of IL-6/JAK/STAT3 signaling [101]; however, whether HSP modulation contributes to this effect remains unclear. Similarly, an injectable hydrogel co-delivering epigallocatechin gallate and recombinant TGF-β has been evaluated in a rat-tail disc degeneration model [102], but the specific contribution of HSP90 regulation requires direct verification. These examples support the broader feasibility of combination therapy but do not yet establish that simultaneous regulation of protective and pathogenic HSP pathways produces superior outcomes.

Major translational barriers remain. Most HSP-related IVDD studies have been conducted in isolated cells or rodent injury models, which do not fully reproduce the size, nutritional environment, biomechanical loading, and chronic progression of human disc degeneration. Large-animal studies should therefore assess local pharmacokinetics, treatment durability, dose–response relationships, tissue compatibility, material degradation, immunogenicity, and long-term biomechanical safety. Mechanistic studies should include HSP-specific inhibition, knockdown, rescue, or isoform-selective intervention groups to determine whether observed benefits are causally dependent on the proposed HSP pathway. Human disc explants and well-characterized patient tissues may provide an intermediate platform for evaluating biological relevance before clinical investigation.

Finally, integration with existing treatments should be considered cautiously. Local biomaterial delivery after annulus fibrosus repair or discectomy may offer a means of retaining HSP-modulating agents within the disc, whereas physical interventions such as MES remain hypothesis-generating because direct IVDD-specific HSP evidence is lacking [93]. Future studies should prioritize reproducible manufacturing, minimally disruptive delivery, clinically relevant outcome measures, and comparison with current standards of care. Collectively, these efforts may clarify the feasibility, safety, and therapeutic relevance of HSP-targeted interventions and support their further preclinical evaluation.

## 6. Conclusions

IVDD involves progressive disruption of extracellular matrix homeostasis, cellular stress adaptation, inflammatory signaling, senescence, autophagy, and programmed cell death. HSPs constitute an important component of the proteostasis network underlying these processes, but their functions are highly context dependent. Intracellular HSP70 family members and HSPA8-mediated chaperone-mediated autophagy generally support cellular survival and protein quality control, whereas extracellular HSP70 may amplify inflammatory signaling. GRP78/HSPA5 participates in both adaptive and maladaptive endoplasmic reticulum stress responses, while HSP90 may support metabolic adaptation under some conditions but stabilize inflammatory, necroptotic, fibrotic, and angiogenic pathways during chronic degeneration. Evidence for HSP40 and HSP60 in IVDD remains comparatively limited and is predominantly indirect.

HSP-centered modulation therefore represents an emerging preclinical framework rather than an established treatment for IVDD. Pharmacological modulation, mild photothermal therapy, natural compounds, physical stimulation, biomaterial-based delivery, and stem cell preconditioning provide potential experimental approaches, but their evidence levels and dependence on specific HSP mechanisms vary considerably. Future research should define the spatiotemporal and isoform-specific functions of HSPs, establish causal mechanisms in disc-specific models, and evaluate local delivery, long-term safety, and biomechanical compatibility in large-animal and human tissue studies. These efforts may facilitate the development and preclinical evaluation of more precise proteostasis-oriented interventions for IVDD.

## Figures and Tables

**Table 1:** Summary of the principal heat shock protein family members implicated in intervertebral disc degeneration.

HSP Family/Isoform	Disc Cell or Tissue Type	Experimental Model	Principal Pathway or Mechanism	Biological Effect in IVDD	Evidence Category
**HSP27/HSPB1**	Nucleus pulposus cells and cartilage endplate chondrocytes	Human developmental and degenerative intervertebral disc tissues	Age- and development-related changes in HSP27 localization and expression	Potential involvement in cellular stress adaptation and cytoprotection	Direct human tissue evidence, but observational and mechanistically limited [46]
**HSP27/HSPB1**	No disc-specific cell type directly validated for the proposed mechanisms	Mechanistic studies in non-IVDD cellular and disease models	p38 MAPK/MK2-dependent phosphorylation; cytoskeletal regulation; PI3K/Akt signaling; potential modulation of apoptosis and ferroptosis	Proposed stabilization of the cytoskeleton and protection against apoptosis and ferroptosis	Indirect, hypothesis-generating evidence extrapolated from non-IVDD models [43,44,45,46,47,48,49,51]
**HSP40/DNAJB9**	Intervertebral disc transcriptomic datasets; no disc-specific experimental validation	Bioinformatic analysis of GSE150408 and GSE124272, supported by general molecular-chaperone studies	HSP70 co-chaperone activity; protein quality control; mitochondrial protein import and biogenesis; autophagy-related gene network	Potential association with proteostasis, autophagy dysregulation, and IVDD progression	Bioinformatic and indirect mechanistic evidence; no disc-specific functional validation [1,52,53,54,55]
**HSP60/HSPD1**	No disc-specific cell type directly validated	Heart failure, COPD, and immune-related disease models	Mitochondrial protein folding; TLR4/MyD88/NF-κB signaling; NLRP3 inflammasome activation	Potential regulation of mitochondrial dysfunction, apoptosis, and inflammation	Indirect evidence from non-IVDD models; direct IVDD-specific evidence is lacking [39,40,41]
**HSP70; isoform not specified**	Nucleus pulposus cells	Compression-induced cellular injury model	SIRT3/Fis1/OPA1 axis; regulation of mitochondrial fission and ROS	Attenuation of mitochondrial dysfunction and compression-induced apoptosis	Direct disc-specific in vitro evidence [9]
**HSPA8/Hsc70**	Human nucleus pulposus tissues and nucleus pulposus cells	Human tissue expression analysis and cellular senescence models	UCHL1-mediated stabilization of HSPA8; HSPA8-dependent chaperone-mediated autophagy and lysosomal degradation of HPCAL1	Promotion of chaperone-mediated autophagy and attenuation of nucleus pulposus cell senescence	Direct human tissue-associated and in vitro mechanistic evidence; no clinical interventional evidence [34,59]
**HSP70; isoform not specified**	Human nucleus pulposus-derived stem/progenitor cells	t-BHP-induced oxidative-stress model; pharmacological activation with TRC051384	Inhibition of JNK/c-Jun and p53/p21 signaling; reduction of ROS; preservation of mitochondrial membrane potential and ATP levels	Attenuation of oxidative stress-induced apoptosis and senescence	Direct disc-specific in vitro evidence; in vivo validation remains limited [60]
**Extracellular HSP70**	Direct disc-cell targets have not been sufficiently validated	Non-IVDD inflammatory and autoimmune disease models	Putative DAMP-mediated activation of TLR2/4–NF-κB signaling	Potential amplification of inflammatory cytokine production within the degenerative disc microenvironment	Indirect, hypothesis-generating evidence extrapolated from non-IVDD models; direct IVDD validation remains limited [64,65]
**GRP78/HSPA5/BiP**	Nucleus pulposus cells	IL-1β-induced degeneration model; parecoxib treatment	COX-2/PGE2–GRP78–CHOP–caspase-12-associated ER-stress signaling	Increased apoptosis and extracellular matrix degradation; attenuation by parecoxib	Direct disc-specific in vitro evidence; GRP78-specific causality requires further validation [35]
**GRP78/HSPA5/BiP**	Nucleus pulposus cells	IL-1β stimulation and 5-azacytidine treatment	DNMT1/DNMT3a-mediated PPARγ promoter methylation and GRP78-associated ER stress	Regulation of ER stress and nucleus pulposus cell apoptosis	Direct disc-specific in vitro evidence [67]
**GRP78/HSPA5/BiP**	Human nucleus pulposus cells and degenerative disc tissues	Increased extracellular matrix-stiffness model	Piezo1 activation, calcium overload, ROS accumulation, and GRP78-associated ER stress	Promotion of cellular senescence and apoptosis under a stiffened matrix microenvironment	Direct human tissue-associated and in vitro evidence; GRP78-specific causality remains unresolved [68]
**GRP78/HSPA5/BiP**	Human annulus fibrosus cells	High cyclic-stretch model	GRP78-associated ER stress; NOX2/ROS/TXNIP/NLRP3 inflammasome signaling	Increased IL-1β release, inflammation, and extracellular matrix degradation	Direct human annulus fibrosus cell evidence in vitro; GRP78-specific causality remains to be established [36]
**GRP78/HSPA5/BiP**	Human nucleus pulposus tissues and nucleus pulposus cells	Human degenerative disc tissue analysis and TNF-α-stimulated human nucleus pulposus cell model	GRP78-associated circ-4099 transcription; circ-4099/miR-616-5p/Sox9 axis	Increased aggrecan and collagen II expression, suggesting a context-dependent compensatory ECM-protective response	Direct human tissue-associated and in vitro mechanistic evidence; in vivo validation remains lacking [70]
**HSP90; isoform not specified**	Nucleus pulposus cells	Advanced glycation end-product exposure	Competition between HSP90 and RACK1 for HIF-1α binding; regulation of HIF-1α and GLUT1/3	Maintenance of hypoxic metabolic adaptation and glucose metabolism	Direct disc-specific in vitro mechanistic evidence [37]
**HSP90; isoform not specified**	Nucleus pulposus cells	M1-polarized macrophage-conditioned medium; treatment with 17-AAG	NF-κB and JAK2/STAT3 signaling; compensatory induction of HSP70	HSP90 inhibition reduces inflammation, matrix catabolism, and inflammatory signaling	Direct disc-specific in vitro evidence [10]
**HSP90; isoform not specified**	Nucleus pulposus-derived stem/progenitor cells	Compression-induced injury; treatment with BIIB021	RIPK1/RIPK3/MLKL signaling; mitochondrial dysfunction; compensatory HSP70 induction	HSP90 inhibition reduces necroptosis and apoptosis	Direct disc-specific in vitro evidence [13]
**HSP90; isoform not specified**	Nucleus pulposus cells	Bone marrow-derived macrophage-conditioned medium; treatment with 17-AAG	CEMIP and RhoA/ROCK/AKT signaling	Attenuation of fibrotic transformation and pathological angiogenic responses following HSP90 inhibition	Direct disc-specific preclinical evidence, predominantly in vitro; translational validation remains limited [38]

**Abbreviations:** COPD, chronic obstructive pulmonary disease; DAMP, damage-associated molecular pattern; ECM, extracellular matrix; ER, endoplasmic reticulum; IVDD, intervertebral disc degeneration; ROS, reactive oxygen species; t-BHP, tert-butyl hydroperoxide. Evidence categories were assigned descriptively according to whether the supporting findings were derived from human disc tissues, disc-specific in vitro studies, bioinformatic analyses, or mechanistic studies conducted in non-IVDD models. These categories do not represent a formal risk-of-bias or GRADE assessment.

## Data Availability

No new data were created or analyzed in this study. Data sharing is not applicable to this article.

## Abbreviations

IVDD	Intervertebral disc degeneration
HSP	Heat Shock Protein
HIF	Hypoxia-inducible factor
SASP	Senescence-associated secretory phenotype
NTD	N-terminal domain
CTD	C-terminal domain
ACD	A-crystallin domain
NP	Nucleus pulposus
NPCs	Nucleus pulposus cells
BMDM-CM	Bone marrow-derived macrophage-conditioned medium
COPD	Chronic obstructive pulmonary disease
ROS	Reactive oxygen species
AGEs	Advanced glycation end products
TAT	Trans-activator of transcription
COX-2	Cyclooxygenase-2
PGE2	Prostaglandin E2
MPTT	Mild photothermal therapy
MES	Mild electrical stimulation
BMSCs	Bone marrow-derived mesenchymal stem cells
